# Methyl 3-[(6-nitro-4-oxo-3-phenyl-3,4-di­hydro­quinazolin-2-yl)sulfan­yl]propano­ate

**DOI:** 10.1107/S1600536813016127

**Published:** 2013-06-19

**Authors:** Ibrahim A. Al-Suwaidan, Alaa A.-M. Abdel-Aziz, Adel S. El-Azab, C. S. Chidan Kumar, Hoong-Kun Fun

**Affiliations:** a Department of Pharmaceutical Chemistry, College of Pharmacy, King Saud University, Riyadh 11451, Saudi Arabia; b Department of Medicinal Chemistry., Faculty of Pharmacy, University of Mansoura, Mansoura 35516, Egypt; c Department of Organic Chemistry, Faculty of Pharmacy, Al-Azhar University, Cairo 11884, Egypt; d X-ray Crystallography Unit, School of Physics, Universiti Sains Malaysia, 11800 USM, Penang, Malaysia

## Abstract

In the title compound, C_18_H_15_N_3_O_5_S, the approximately planar quinazoline ring system [maximum deviation = 0.097 (3) Å] forms a dihedral angle of 76.53 (19)° with the phenyl ring. The terminal -C(=O)—O—C group is disordered over two sets of sites with a site-occupancy ratio of 0.811 (17):0.189 (17). In the crystal, mol­ecules are linked *via* weak C—H⋯O hydrogen bonds into sheets parallel to the *ac* plane.

## Related literature

For background to quinazoline chemistry, see: El-Azab (2007 ▶); El-Azab *et al.* (2010 ▶, 2011 ▶); Alafeefy *et al.* (2008 ▶); Al-Suwaidan *et al.* (2013 ▶); El-Azab & ElTahir (2012*a* ▶,*b* ▶). For standard bond-length data, see: Allen *et al.* (1987 ▶).
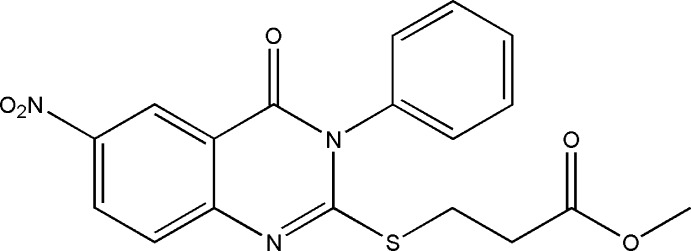


## Experimental

### 

#### Crystal data

C_18_H_15_N_3_O_5_S
*M* *_r_* = 385.39
Monoclinic, 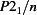

*a* = 4.9146 (3) Å
*b* = 26.5065 (18) Å
*c* = 14.0900 (9) Å
β = 94.645 (4)°
*V* = 1829.5 (2) Å^3^
*Z* = 4
Cu *K*α radiation
μ = 1.89 mm^−1^
*T* = 296 K
0.32 × 0.26 × 0.13 mm

#### Data collection

Bruker SMART APEXII CCD area-detector diffractometer
Absorption correction: multi-scan (*SADABS*; Bruker, 2009 ▶) *T*_min_ = 0.583, *T*_max_ = 0.791
12669 measured reflections
3382 independent reflections
1861 reflections with *I* > 2σ(*I*)
*R*_int_ = 0.059

#### Refinement

*R*[*F* ^2^ > 2σ(*F* ^2^)] = 0.064
*wR*(*F* ^2^) = 0.191
*S* = 1.03
3382 reflections
273 parameters
9 restraints
H-atom parameters constrained
Δρ_max_ = 0.24 e Å^−3^
Δρ_min_ = −0.16 e Å^−3^

### 

Data collection: *APEX2* (Bruker, 2009 ▶); cell refinement: *SAINT* (Bruker, 2009 ▶); data reduction: *SAINT*; program(s) used to solve structure: *SHELXTL* (Sheldrick, 2008 ▶); program(s) used to refine structure: *SHELXTL*; molecular graphics: *SHELXTL*; software used to prepare material for publication: *SHELXTL* and *PLATON* (Spek, 2009 ▶).

## Supplementary Material

Crystal structure: contains datablock(s) global, I. DOI: 10.1107/S1600536813016127/lh5622sup1.cif

Structure factors: contains datablock(s) I. DOI: 10.1107/S1600536813016127/lh5622Isup2.hkl

Supplementary material file. DOI: 10.1107/S1600536813016127/lh5622Isup3.cml

Additional supplementary materials:  crystallographic information; 3D view; checkCIF report

## Figures and Tables

**Table 1:** Hydrogen-bond geometry (Å, °)

*D*—H⋯*A*	*D*—H	H⋯*A*	*D*⋯*A*	*D*—H⋯*A*
C6—H6*A*⋯O2^i^	0.93	2.56	3.142 (5)	121
C10—H10*A*⋯O2^ii^	0.93	2.39	3.167 (5)	140

Symmetry codes: (i) 
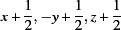
; (ii) 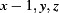.

**Fig. 1.**
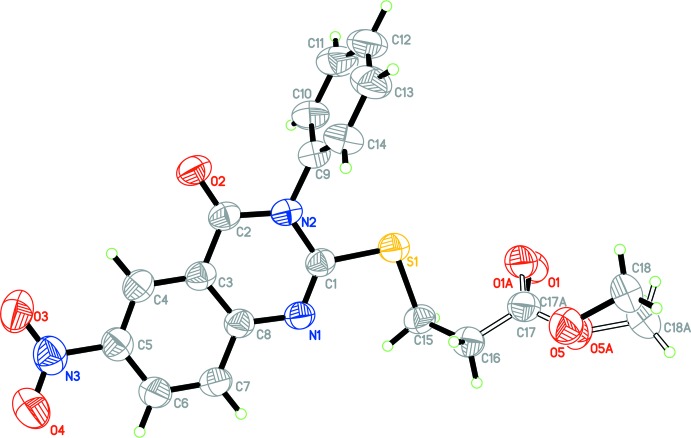
The molecular structure of the title compound with 30% probability displacement ellipsoids. The minor component of disorder is shown as open bonds

**Fig. 2.**
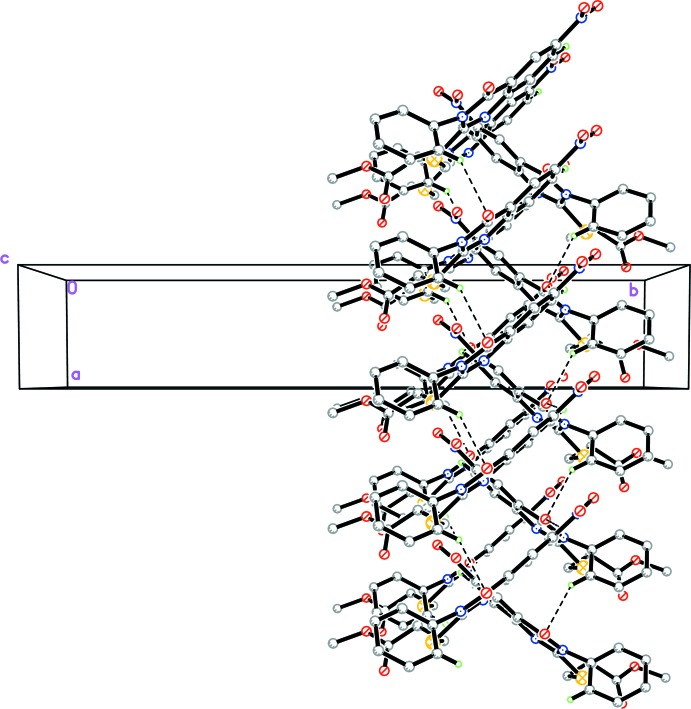
The crystal packing of the title compound. The minor component of disorder and H atoms not involved in the intermolecular interactions (dashed lines) have been omitted for clarity.

## supplementary crystallographic information

### Comment

Quinazolines are considered to be important chemical synthons of physiological significance and pharmaceutical utility. They possess a variety of biological effects including antimicrobial (El-Azab *et al.*, 2007), anti-inflammatory (Alafeefy *et al.*, 2008), anticonvulsant, (El-Azab *et al.*, 2011; El-Azab & ElTahir 2012*a*,*b*) and anticancer activities (El-Azab *et al.*, 2010; El-Azab & ElTahir 2012*b*; Al-Suwaidan *et al.*, 2013). These observations have been the guidelines for the development of new quinazolines which possess varied biological activities. Prompted by the potential biological activities of quinazolines, the title compound was synthesized and its crystal structure is reported herein.

The molecular structure of the title compound is shown in Fig. 1. The quinazoline ring (C1–C8/N1–N2; maximum deviation = 0.097 (3) Å at atom N2) makes a dihedral angle of 76.53 (19)° with the attached phenyl ring (C9–C14). The terminal C17(═O1)—O5—C18 group is disordered over two positions with a site-occupancy ratio of 0.811 (17): 0.189 (17). In the crystal structure (Fig. 2), the molecules are linked *via* weak intermolecular C6—H6A···O2^i^ and C10—H10A···O2^ii^ hydrogen bonds (see Table 1 for symmetry codes) into sheets parallel to the *ac* plane.

### Experimental

A mixture of 3-phenyl-2-mercapto-6-nitro-quinazolin-4(3*H*)-one (2.99 g, 0.01 mol) and Et_3_N (2 ml) in CH_2_Cl_2_ (30 ml) was stirred in an ice bath and acryloyl chloride (3.3 ml, 0.04 mol) was added dropwise over a period of 15 min. Stirring was performed in an ice bath for 1 h and then at room temperature overnight. Solvent was then removed under reduced pressure and the obtained residue was dissolved in CH_2_Cl_2_ and washed with 10% NaOH solution and water. The resultant was separated and dried over MgSO_4_ then evaporated *in vacuo*. The obtained residue was chromatographed on silica gel using 10% EtOAc/hexane as eluant and recrystallized from hexane/CH_2_Cl_2_ (92%) (m.p:. 480 K) to yield X-ray quality crystals.

### Refinement

The hydrogen atoms were positioned geometrically [C–H = 0.93, 0.96 and 0.97 Å] and were refined using a riding model, with *U*_iso_(H) = 1.2 *U*_eq_(C) or 1.5*U*_eq_(methyl C). A rotating-group model was used for the methyl groups. The terminal C17(═O1)—O5—C18 group is disordered over two positions with a site-occupancy ratio of 0.811 (17): 0.189 (17). The SHELXL (Sheldrick, 2008) EXYZ (same *x*, *y* and *z* parameters) and EADP (same *U*_ij_ parameters) restraints were used for atoms pairs C17/C17A.The same distance restraints were applied to (C17/O1 & C17A/O1A), (C17/O5 & C17A/O5A) and (C18/O5 & C18A/O5A). The SIMU (similar *U*_ij_ parameters) restraint was applied to C18/C18A.

### Crystal data

**Table:**

C_18_H_15_N_3_O_5_S	*F*(000) = 800
*M**_r_* = 385.39	*D*_x_ = 1.399 Mg m^−^^3^
Monoclinic, *P*2_1_/*n*	Melting point: 480 K
Hall symbol: -P 2yn	Cu *K*α radiation, λ = 1.54178 Å
*a* = 4.9146 (3) Å	Cell parameters from 1209 reflections
*b* = 26.5065 (18) Å	θ = 3.3–67.5°
*c* = 14.0900 (9) Å	µ = 1.89 mm^−^^1^
β = 94.645 (4)°	*T* = 296 K
*V* = 1829.5 (2) Å^3^	Block, colourless
*Z* = 4	0.32 × 0.26 × 0.13 mm

### Data collection

**Table:**

Bruker SMART APEXII CCD area-detector diffractometer	3382 independent reflections
Radiation source: fine-focus sealed tube	1861 reflections with *I* > 2σ(*I*)
Graphite monochromator	*R*_int_ = 0.059
φ and ω scans	θ_max_ = 69.8°, θ_min_ = 3.3°
Absorption correction: multi-scan (*SADABS*; Bruker, 2009)	*h* = −5→4
*T*_min_ = 0.583, *T*_max_ = 0.791	*k* = −31→31
12669 measured reflections	*l* = −17→17

### Refinement

**Table:**

Refinement on *F*^2^	Secondary atom site location: difference Fourier map
Least-squares matrix: full	Hydrogen site location: inferred from neighbouring sites
*R*[*F*^2^ > 2σ(*F*^2^)] = 0.064	H-atom parameters constrained
*wR*(*F*^2^) = 0.191	*w* = 1/[σ^2^(*F*_o_^2^) + (0.0825*P*)^2^ + 0.1932*P*] where *P* = (*F*_o_^2^ + 2*F*_c_^2^)/3
*S* = 1.03	(Δ/σ)_max_ < 0.001
3382 reflections	Δρ_max_ = 0.24 e Å^−^^3^
273 parameters	Δρ_min_ = −0.16 e Å^−^^3^
9 restraints	Extinction correction: *SHELXTL* (Sheldrick, 2008), Fc^*^=kFc[1+0.001xFc^2^λ^3^/sin(2θ)]^-1/4^
Primary atom site location: structure-invariant direct methods	Extinction coefficient: 0.0030 (5)

### Special details

**Table:**

Geometry. All e.s.d.'s (except the e.s.d. in the dihedral angle between two l.s. planes) are estimated using the full covariance matrix. The cell e.s.d.'s are taken into account individually in the estimation of e.s.d.'s in distances, angles and torsion angles; correlations between e.s.d.'s in cell parameters are only used when they are defined by crystal symmetry. An approximate (isotropic) treatment of cell e.s.d.'s is used for estimating e.s.d.'s involving l.s. planes.
Refinement. Refinement of *F*^2^ against ALL reflections. The weighted *R*-factor *wR* and goodness of fit *S* are based on *F*^2^, conventional *R*-factors *R* are based on *F*, with *F* set to zero for negative *F*^2^. The threshold expression of *F*^2^ > σ(*F*^2^) is used only for calculating *R*-factors(gt) *etc*. and is not relevant to the choice of reflections for refinement. *R*-factors based on *F*^2^ are statistically about twice as large as those based on *F*, and *R*- factors based on ALL data will be even larger.

### Fractional atomic coordinates and isotropic or equivalent isotropic displacement parameters (Å^2^)

**Table:**

	*x*	*y*	*z*	*U*_iso_*/*U*_eq_	Occ. (<1)
S1	0.3830 (2)	0.12146 (4)	0.63782 (7)	0.0880 (4)	
O2	0.8688 (6)	0.19581 (12)	0.38141 (18)	0.0982 (9)	
O3	1.5168 (8)	0.33688 (17)	0.4490 (3)	0.1442 (16)	
O4	1.6007 (9)	0.36422 (17)	0.5910 (3)	0.1520 (16)	
N1	0.7309 (6)	0.19860 (13)	0.6615 (2)	0.0790 (9)	
N2	0.6827 (6)	0.16072 (12)	0.50857 (19)	0.0741 (8)	
N3	1.4799 (8)	0.33704 (17)	0.5326 (4)	0.1094 (13)	
C1	0.6245 (7)	0.16499 (15)	0.6034 (2)	0.0738 (9)	
C2	0.8395 (7)	0.19721 (16)	0.4673 (3)	0.0786 (10)	
C3	0.9676 (7)	0.23374 (15)	0.5321 (2)	0.0760 (10)	
C4	1.1534 (8)	0.26847 (16)	0.5010 (3)	0.0849 (11)	
H4A	1.1912	0.2694	0.4374	0.102*	
C5	1.2796 (8)	0.30119 (17)	0.5656 (3)	0.0884 (11)	
C6	1.2314 (9)	0.30066 (18)	0.6598 (3)	0.0984 (13)	
H6A	1.3229	0.3229	0.7023	0.118*	
C7	1.0479 (9)	0.26718 (17)	0.6906 (3)	0.0927 (12)	
H7A	1.0117	0.2671	0.7544	0.111*	
C8	0.9117 (7)	0.23265 (15)	0.6269 (3)	0.0787 (10)	
C9	0.5906 (7)	0.11756 (17)	0.4514 (2)	0.0783 (10)	
C10	0.3811 (8)	0.1233 (2)	0.3819 (3)	0.0991 (14)	
H10A	0.2985	0.1545	0.3706	0.119*	
C11	0.2955 (11)	0.0814 (3)	0.3288 (4)	0.1243 (19)	
H11A	0.1516	0.0844	0.2820	0.149*	
C12	0.4181 (12)	0.0363 (3)	0.3440 (4)	0.1245 (19)	
H12A	0.3567	0.0084	0.3085	0.149*	
C13	0.6334 (11)	0.0314 (2)	0.4119 (4)	0.1198 (17)	
H13A	0.7208	0.0004	0.4212	0.144*	
C14	0.7186 (9)	0.07209 (18)	0.4656 (3)	0.1008 (14)	
H14A	0.8636	0.0689	0.5118	0.121*	
C15	0.3526 (9)	0.14022 (17)	0.7595 (2)	0.0889 (12)	
H15A	0.4555	0.1710	0.7724	0.107*	
H15B	0.1625	0.1473	0.7682	0.107*	
C16	0.4560 (8)	0.09993 (17)	0.8302 (3)	0.0871 (11)	
H16A	0.6427	0.0916	0.8188	0.105*	
H16B	0.4572	0.1136	0.8941	0.105*	
C17	0.2895 (9)	0.05291 (19)	0.8246 (3)	0.0925 (13)	0.811 (17)
O1	0.085 (3)	0.0464 (8)	0.7732 (11)	0.107 (4)	0.811 (17)
O5	0.4116 (19)	0.0176 (3)	0.8814 (6)	0.118 (3)	0.811 (17)
C18	0.264 (3)	−0.0316 (3)	0.8826 (7)	0.157 (4)	0.811 (17)
H18A	0.3690	−0.0551	0.9223	0.235*	0.811 (17)
H18B	0.2381	−0.0447	0.8190	0.235*	0.811 (17)
H18C	0.0892	−0.0266	0.9072	0.235*	0.811 (17)
C17A	0.2895 (9)	0.05291 (19)	0.8246 (3)	0.0925 (13)	0.189 (17)
O1A	0.120 (14)	0.048 (4)	0.759 (5)	0.115 (18)	0.189 (17)
O5A	0.293 (6)	0.0223 (10)	0.9005 (15)	0.089 (7)	0.189 (17)
C18A	0.134 (10)	−0.0214 (14)	0.934 (3)	0.156 (7)	0.189 (17)
H18D	0.2131	−0.0324	0.9952	0.234*	0.189 (17)
H18E	0.1376	−0.0486	0.8893	0.234*	0.189 (17)
H18F	−0.0517	−0.0112	0.9396	0.234*	0.189 (17)

### Atomic displacement parameters (Å^2^)

**Table:**

	*U*^11^	*U*^22^	*U*^33^	*U*^12^	*U*^13^	*U*^23^
S1	0.0921 (7)	0.0934 (8)	0.0790 (6)	−0.0061 (5)	0.0095 (4)	−0.0080 (6)
O2	0.119 (2)	0.109 (3)	0.0674 (15)	0.0031 (17)	0.0072 (13)	−0.0014 (16)
O3	0.148 (3)	0.169 (4)	0.119 (3)	−0.039 (3)	0.023 (2)	0.034 (3)
O4	0.163 (4)	0.138 (4)	0.153 (3)	−0.056 (3)	−0.003 (3)	0.003 (3)
N1	0.092 (2)	0.077 (2)	0.0678 (17)	0.0000 (16)	0.0039 (14)	−0.0049 (17)
N2	0.0814 (19)	0.078 (2)	0.0622 (16)	0.0074 (15)	0.0006 (13)	−0.0056 (16)
N3	0.103 (3)	0.103 (3)	0.120 (3)	−0.006 (2)	−0.004 (2)	0.024 (3)
C1	0.080 (2)	0.073 (3)	0.068 (2)	0.0073 (17)	0.0035 (15)	0.001 (2)
C2	0.086 (2)	0.083 (3)	0.066 (2)	0.012 (2)	−0.0007 (17)	0.001 (2)
C3	0.088 (2)	0.071 (3)	0.068 (2)	0.0111 (19)	0.0005 (16)	0.0045 (19)
C4	0.090 (3)	0.085 (3)	0.079 (2)	0.010 (2)	0.0033 (19)	0.013 (2)
C5	0.092 (3)	0.080 (3)	0.092 (3)	0.002 (2)	−0.002 (2)	0.012 (2)
C6	0.117 (3)	0.089 (3)	0.088 (3)	−0.016 (3)	−0.006 (2)	0.001 (3)
C7	0.116 (3)	0.091 (3)	0.071 (2)	−0.007 (2)	0.003 (2)	−0.004 (2)
C8	0.090 (2)	0.072 (3)	0.073 (2)	0.0019 (19)	0.0002 (17)	0.001 (2)
C9	0.082 (2)	0.086 (3)	0.067 (2)	0.004 (2)	0.0031 (16)	−0.007 (2)
C10	0.095 (3)	0.115 (4)	0.085 (3)	0.009 (2)	−0.010 (2)	−0.016 (3)
C11	0.111 (4)	0.161 (6)	0.098 (3)	−0.008 (4)	−0.015 (3)	−0.030 (4)
C12	0.129 (4)	0.137 (6)	0.108 (4)	−0.031 (4)	0.012 (3)	−0.049 (4)
C13	0.136 (4)	0.095 (4)	0.130 (4)	0.000 (3)	0.016 (3)	−0.038 (3)
C14	0.104 (3)	0.095 (4)	0.100 (3)	0.006 (3)	−0.008 (2)	−0.026 (3)
C15	0.100 (3)	0.093 (3)	0.076 (2)	0.003 (2)	0.0171 (19)	−0.008 (2)
C16	0.082 (2)	0.094 (3)	0.085 (2)	−0.001 (2)	0.0058 (19)	−0.007 (2)
C17	0.092 (3)	0.102 (4)	0.083 (3)	−0.011 (2)	0.005 (2)	−0.009 (3)
O1	0.089 (5)	0.119 (7)	0.110 (6)	−0.015 (5)	−0.006 (6)	−0.006 (5)
O5	0.132 (6)	0.111 (4)	0.109 (4)	−0.017 (4)	−0.014 (4)	0.020 (3)
C18	0.229 (11)	0.110 (6)	0.130 (7)	−0.069 (7)	0.011 (6)	0.006 (5)
C17A	0.092 (3)	0.102 (4)	0.083 (3)	−0.011 (2)	0.005 (2)	−0.009 (3)
O1A	0.10 (3)	0.14 (3)	0.10 (2)	−0.05 (2)	0.028 (16)	−0.02 (2)
O5A	0.083 (15)	0.101 (15)	0.082 (12)	−0.005 (11)	−0.005 (9)	0.027 (10)
C18A	0.226 (15)	0.103 (11)	0.137 (12)	−0.061 (11)	0.004 (11)	−0.006 (11)

### Geometric parameters (Å, º)

**Table:**

S1—C1	1.752 (4)	C10—H10A	0.9300
S1—C15	1.803 (4)	C11—C12	1.348 (7)
O2—C2	1.231 (4)	C11—H11A	0.9300
O3—N3	1.207 (5)	C12—C13	1.374 (7)
O4—N3	1.212 (5)	C12—H12A	0.9300
N1—C1	1.291 (4)	C13—C14	1.364 (6)
N1—C8	1.382 (5)	C13—H13A	0.9300
N2—C2	1.393 (5)	C14—H14A	0.9300
N2—C1	1.394 (4)	C15—C16	1.519 (5)
N2—C9	1.451 (5)	C15—H15A	0.9700
N3—C5	1.470 (6)	C15—H15B	0.9700
C2—C3	1.440 (5)	C16—C17	1.489 (6)
C3—C8	1.386 (5)	C16—H16A	0.9700
C3—C4	1.393 (5)	C16—H16B	0.9700
C4—C5	1.369 (5)	C17—O1	1.204 (7)
C4—H4A	0.9300	C17—O5	1.342 (6)
C5—C6	1.367 (6)	O5—C18	1.494 (8)
C6—C7	1.361 (6)	C18—H18A	0.9600
C6—H6A	0.9300	C18—H18B	0.9600
C7—C8	1.412 (5)	C18—H18C	0.9600
C7—H7A	0.9300	O5A—C18A	1.496 (9)
C9—C14	1.367 (6)	C18A—H18D	0.9600
C9—C10	1.371 (5)	C18A—H18E	0.9600
C10—C11	1.385 (7)	C18A—H18F	0.9600
			
C1—S1—C15	101.0 (2)	C12—C11—H11A	119.6
C1—N1—C8	117.8 (3)	C10—C11—H11A	119.6
C2—N2—C1	120.5 (3)	C11—C12—C13	120.3 (5)
C2—N2—C9	118.3 (3)	C11—C12—H12A	119.9
C1—N2—C9	121.2 (3)	C13—C12—H12A	119.9
O3—N3—O4	124.1 (5)	C14—C13—C12	119.7 (5)
O3—N3—C5	117.6 (5)	C14—C13—H13A	120.1
O4—N3—C5	118.3 (5)	C12—C13—H13A	120.1
N1—C1—N2	124.0 (4)	C13—C14—C9	120.0 (4)
N1—C1—S1	121.9 (3)	C13—C14—H14A	120.0
N2—C1—S1	114.1 (3)	C9—C14—H14A	120.0
O2—C2—N2	120.1 (4)	C16—C15—S1	112.3 (3)
O2—C2—C3	124.3 (4)	C16—C15—H15A	109.1
N2—C2—C3	115.5 (3)	S1—C15—H15A	109.1
C8—C3—C4	120.3 (4)	C16—C15—H15B	109.1
C8—C3—C2	119.1 (4)	S1—C15—H15B	109.1
C4—C3—C2	120.6 (3)	H15A—C15—H15B	107.9
C5—C4—C3	118.9 (4)	C17—C16—C15	113.6 (4)
C5—C4—H4A	120.6	C17—C16—H16A	108.8
C3—C4—H4A	120.6	C15—C16—H16A	108.8
C6—C5—C4	122.3 (4)	C17—C16—H16B	108.8
C6—C5—N3	119.1 (4)	C15—C16—H16B	108.8
C4—C5—N3	118.6 (4)	H16A—C16—H16B	107.7
C7—C6—C5	119.2 (4)	O1—C17—O5	124.8 (11)
C7—C6—H6A	120.4	O1—C17—C16	125.5 (11)
C5—C6—H6A	120.4	O5—C17—C16	109.6 (5)
C6—C7—C8	120.8 (4)	C17—O5—C18	114.9 (7)
C6—C7—H7A	119.6	O5—C18—H18A	109.5
C8—C7—H7A	119.6	O5—C18—H18B	109.5
N1—C8—C3	122.4 (3)	H18A—C18—H18B	109.5
N1—C8—C7	119.0 (3)	O5—C18—H18C	109.5
C3—C8—C7	118.6 (4)	H18A—C18—H18C	109.5
C14—C9—C10	120.8 (4)	H18B—C18—H18C	109.5
C14—C9—N2	119.7 (3)	O5A—C18A—H18D	109.5
C10—C9—N2	119.5 (4)	O5A—C18A—H18E	109.5
C9—C10—C11	118.4 (5)	H18D—C18A—H18E	109.5
C9—C10—H10A	120.8	O5A—C18A—H18F	109.5
C11—C10—H10A	120.8	H18D—C18A—H18F	109.5
C12—C11—C10	120.8 (5)	H18E—C18A—H18F	109.5
			
C8—N1—C1—N2	0.6 (5)	C1—N1—C8—C3	4.1 (6)
C8—N1—C1—S1	−177.5 (3)	C1—N1—C8—C7	−174.9 (3)
C2—N2—C1—N1	−7.6 (5)	C4—C3—C8—N1	−179.4 (3)
C9—N2—C1—N1	170.2 (3)	C2—C3—C8—N1	−1.9 (6)
C2—N2—C1—S1	170.6 (3)	C4—C3—C8—C7	−0.3 (6)
C9—N2—C1—S1	−11.6 (4)	C2—C3—C8—C7	177.2 (3)
C15—S1—C1—N1	0.6 (4)	C6—C7—C8—N1	178.7 (4)
C15—S1—C1—N2	−177.6 (3)	C6—C7—C8—C3	−0.4 (6)
C1—N2—C2—O2	−173.2 (3)	C2—N2—C9—C14	103.2 (4)
C9—N2—C2—O2	9.0 (5)	C1—N2—C9—C14	−74.6 (5)
C1—N2—C2—C3	9.3 (5)	C2—N2—C9—C10	−74.9 (5)
C9—N2—C2—C3	−168.6 (3)	C1—N2—C9—C10	107.2 (4)
O2—C2—C3—C8	177.8 (4)	C14—C9—C10—C11	2.4 (7)
N2—C2—C3—C8	−4.8 (5)	N2—C9—C10—C11	−179.5 (4)
O2—C2—C3—C4	−4.7 (6)	C9—C10—C11—C12	−1.0 (8)
N2—C2—C3—C4	172.7 (3)	C10—C11—C12—C13	−1.0 (9)
C8—C3—C4—C5	0.2 (6)	C11—C12—C13—C14	1.7 (8)
C2—C3—C4—C5	−177.3 (3)	C12—C13—C14—C9	−0.3 (8)
C3—C4—C5—C6	0.6 (6)	C10—C9—C14—C13	−1.7 (7)
C3—C4—C5—N3	178.7 (3)	N2—C9—C14—C13	−179.8 (4)
O3—N3—C5—C6	−179.5 (5)	C1—S1—C15—C16	−114.3 (3)
O4—N3—C5—C6	1.7 (7)	S1—C15—C16—C17	−65.9 (4)
O3—N3—C5—C4	2.3 (6)	C15—C16—C17—O1	−3.1 (14)
O4—N3—C5—C4	−176.5 (4)	C15—C16—C17—O5	173.2 (6)
C4—C5—C6—C7	−1.3 (7)	O1—C17—O5—C18	−3.9 (15)
N3—C5—C6—C7	−179.4 (4)	C16—C17—O5—C18	179.8 (6)
C5—C6—C7—C8	1.2 (7)		

### Hydrogen-bond geometry (Å, º)

**Table:**

*D*—H···*A*	*D*—H	H···*A*	*D*···*A*	*D*—H···*A*
C6—H6*A*···O2^i^	0.93	2.56	3.142 (5)	121
C10—H10*A*···O2^ii^	0.93	2.39	3.167 (5)	140

Symmetry codes: (i) *x*+1/2, −*y*+1/2, *z*+1/2; (ii) *x*−1, *y*, *z*.
